# The association between early-life (during pregnancy and after birth) antibiotic exposure and type 1 diabetes: an updated meta-analysis

**DOI:** 10.3389/fendo.2026.1807564

**Published:** 2026-04-22

**Authors:** Yixuan Wu, Hongkui Zhang, Xiaoming Zhang, Kangshuo Hu, Xuelian Dai, Juanjuan Zhu

**Affiliations:** 1 The Second Clinical Medical College of Zhejiang Chinese Medical University, Hangzhou, Zhejiang, China; 2 Pediatrics Department, Yinzhou No. 3 Hospital, Ningbo, Zhejiang, China; 3 The First Clinical Medical College, Zhejiang Chinese Medical University, Hangzhou, Zhejiang, China; 4 Department of Obstetrics and Gynecology, Maternal and Child Health Hospital of Changxing County, Huzhou, Zhejiang, China

**Keywords:** antibiotic, cesarean section, early-life, meta-analysis, pregnancy, type 1 diabetes

## Abstract

**Background:**

The impact of antibiotic exposure on type 1 diabetes (T1D) in childhood or adolescence is controversial. This meta-analysis aimed to explore the association between antibiotic exposure in early life (during pregnancy and after birth) and T1D in children or adolescents.

**Methods:**

We systematically searched for cohort studies on the association between early-life antibiotic exposure and T1D in children or adolescents, in PubMed, Web of Science, EMBASE, and Science Direct from inception to October 2025. Hazard ratios (HRs) and 95% confidence intervals (95%CIs) were calculated to quantify the association between the early-life antibiotic exposure and the risk of T1D in children or adolescents.

**Results:**

Seven studies involving about 7.4 million participants were included in the meta-analysis. Neither the antibiotic exposure during pregnancy (HR = 1.06, 95%CI: 0.98-1.15, p=0.146) nor the antibiotic exposure after birth (HR = 1.03, 95%CI: 0.95-1.11, p=0.491) showed significant differences in the incidence of T1D between the exposed group and the unexposed group. However, a higher incidence of T1D was observed in the exposure after birth compared to unexposed controls among participants born by cesarean section (HR = 1.62, 95%CI: 1.31-2.01, p<0.001). In addition, in subgroup analyzes including sibling comparisons, sex, timing of exposure, frequency of exposure, antibiotic class, and antibacterial spectrum, neither the antibiotic exposure during pregnancy nor the antibiotic exposure after birth found significant differences in the incidence of T1D between both groups.

**Conclusion:**

The study did not find the association between early-life (during pregnancy and after birth) antibiotic exposure and T1D. However, antibiotic exposure after birth might increase the risk of T1D among children born by cesarean section.

## Introduction

Type 1 diabetes mellitus (T1D) is a chronic condition characterized by autoimmune destruction of pancreatic β-cells, necessitating lifelong frequent blood glucose monitoring and regular insulin injections (1). In recent years, the incidence of T1D among children and adolescents has been progressively increasing. Analysis of global data from 1990 to 2021 demonstrated that the prevalence of T1D among children and adolescents increased approximately 1.5-fold across virtually all age subgroups (2). This upward trend cannot be fully explained by genetic susceptibility alone, suggesting that environmental factors, particularly exposures to risk factors like infections, drug exposure and unhealthy lifestyles during critical early life windows, are likely one of the important drivers of the rising incidence. Among these factors, antibiotic use has attracted considerable attention due to its widespread administration during pregnancy and early childhood. A meta-analysis (3) involving 34 million pregnant women from 49 countries indicated that approximately one-quarter of women globally were prescribed antibiotics during pregnancy. Furthermore, a cohort study (4) revealed that more than half of all children received at least one antibiotic prescription by the age of 24 months.

From a biological perspective, antibiotic exposure may profoundly influence early immune system development by disrupting intestinal microbial homeostasis (5), providing a strong theoretical basis for investigating its association with T1D. A cohort study (6) suggested that maternal use of phenoxymethyl penicillins during pregnancy might be associated with an approximately 70% increased risk of developing T1D in childhood. In contrast, meta-analyzes (7, 8) revealed no significant link between antibiotic exposure during early life and the subsequent development of T1D in children. Despite the accumulation of clinical studies, the ongoing debate and conflicting findings underscore the importance of further elucidating the role of early-life antibiotic exposure in T1D development. Based on the above considerations, we conducted an updated meta-analysis of cohort studies, along with comprehensive subgroup analyzes such as sibling analysis, exposure stage, exposure frequency and types of antibiotics, to examine the impact of antibiotic exposure in early life (during pregnancy and after birth) on the risk of T1D during childhood or adolescence.

## Methods

### Study registration

In accordance with the Preferred Reporting Items for Systematic Reviews and Meta-Analyses (PRISMA) guidelines (9), this meta-analysis was prospectively registered on the International Prospective Register of Systematic Reviews (PROSPERO) under the identifier CRD420251231347. A systematic literature search was performed from database inception until October 2025 to investigate the association between early-life antibiotic exposure (during pregnancy and after birth) and the incidence of T1D in children and adolescents. The search was conducted across four electronic databases: PubMed, Web of Science, Embase, and the Cochrane Library. The key search terms employed were: (Antibacterial OR Antibiotic OR Antibiotics OR Antimicrobial OR Anti-infection OR Anti-infective) AND (IA OR T1D OR (Type 1 diabetes) OR (Islet Autoimmunity)). To minimize the risk of omitting eligible studies, the reference lists of all included articles were additionally screened.

### Selection and exclusion criteria

Studies were selected in accordance with the PECOS (Participants, Exposure, Comparison, Outcomes, Study design) framework. Eligible studies satisfied the following inclusion criteria: (1) Participants: children who were free of T1D at the start of follow-up period for T1D assessment; (2) Exposure: exposure to antibiotics on one or more occasions during pregnancy (from the fetal period to the completion of birth) or within the first 24 months after birth (from the completion of birth through the first 24 months of life); (3) Comparison: no exposure to antibiotics during the same periods (pregnancy or the first 24 months of life); (4) Outcomes: outcomes related to the incidence of T1D in children and adolescents under 20 years of age; (5) Study design: only cohort studies.

Studies were excluded if they met any of the following criteria: (1) full text was unavailable; (2) publications were written in a language other than English; (3) relevant outcomes were not reported; (4) accessible data were insufficient or unsuitable for statistical analysis; (5) updated publications, in which case the most recent or comprehensive version was retained.

### Data extraction and quality assessment

Data were extracted systematically by two independent investigators, following a pre-designed and programmed table that gathered essential information including authors, year of publication, participant characteristics (country, year of birth, number of participants, number of T1D, therapeutic regimen of antibiotics, exposure stage, exposure frequency and modes of birth) and adjusted factors.

For cohort studies, the quality was assessed using the Newcastle-Ottawa Scale (NOS). This tool appraises studies across three key domains: participant selection, group comparability, and the ascertainment of outcomes. Studies achieving a score above 6 were deemed to be of high methodological quality. Any discrepancies in the initial ratings were resolved through discussion among the authors until a consensus was reached.

### Statistical analysis

All statistical analyzes were conducted using Stata software version 12.0 (Stata Corporation LLC, College Station, USA). The association between early life antibiotic exposure and the risk of T1D was quantified by calculating hazard ratios (HRs) with corresponding 95% confidence intervals (95%CIs), where an HR>1 was interpreted as evidence that antibiotic exposure is associated with an increased risk of T1D, whereas an HR<1 suggested a potential protective effect against T1D. To evaluate the consistency of findings across studies, heterogeneity was assessed using the chi-square test and quantified with the *I²* statistic; *I²* values below 25% were considered low, 25-50% moderate, and above 50% substantial (10). In this context, heterogeneity mainly consisted of statistical heterogeneity such as varying effect estimates across studies, clinical heterogeneity such as different participant characteristics, and methodological heterogeneity such as different ways of ascertaining outcomes. Given the inherent heterogeneity among the included studies, a random-effects model was applied for meta-analyzes to derive more reliable results. Subgroup analyzes (including sibling analysis, sex, timing of exposure, times of exposure, mode of delivery and types of antibiotics) were conducted to explore potential sources of heterogeneity. Further analyzes included Egger’s test to evaluate publication bias, and sensitivity analyzes performed by sequentially excluding individual studies to examine the robustness of the pooled results. All statistical tests were two-sided, with a p<0.05 considered statistically significant.

## Results

### Study selection

**Figure 1:**
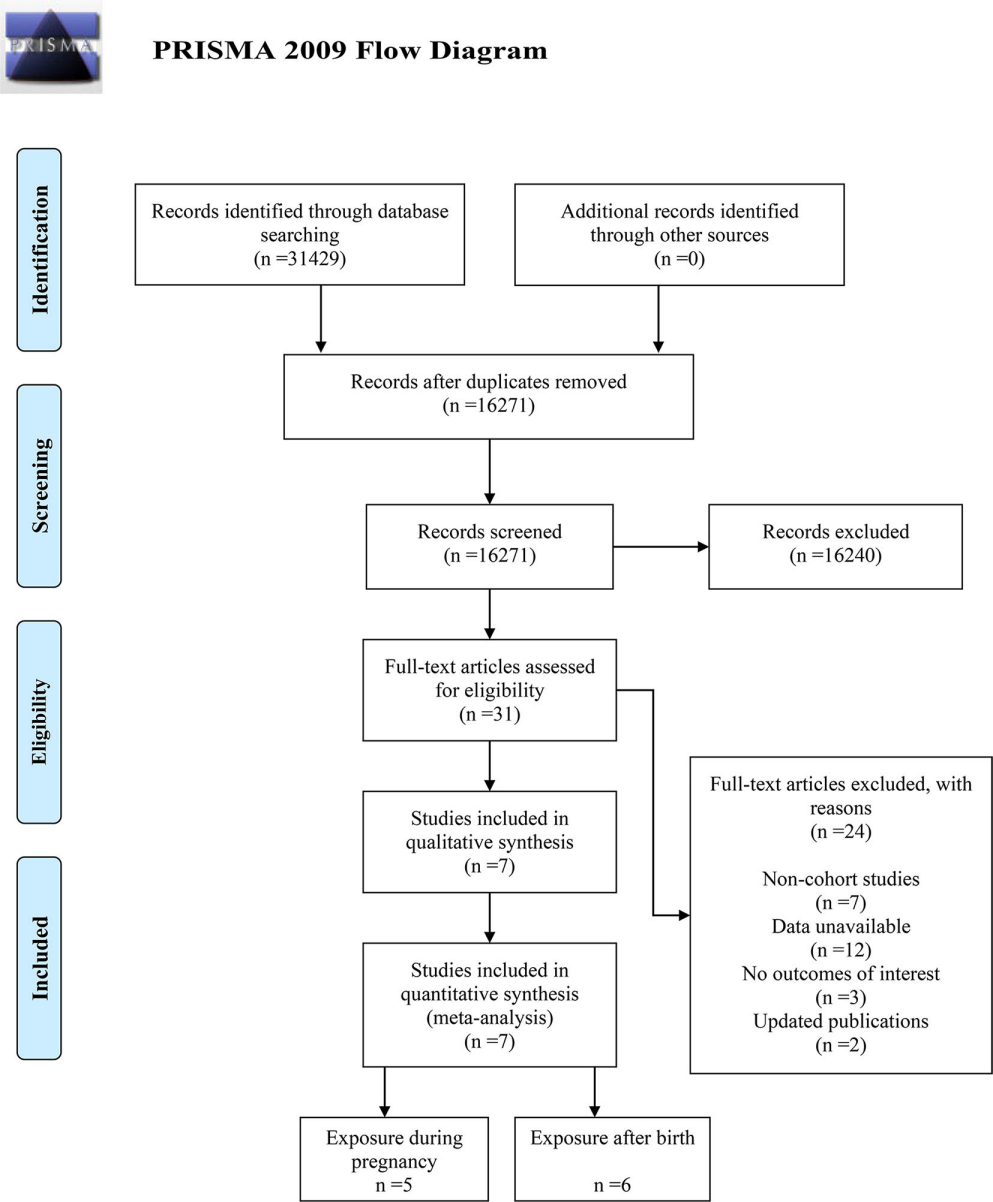
Flow chart of study selection.

A systematic literature search was conducted across four databases using predefined search strategies, yielding a total of 31,429 records. No additional records were identified through reference screening of included studies. After removing duplicates, 16,271 records remained. Following title and abstract screening, 16,240 records were excluded due to little relevance, leaving 31 full-text articles for eligibility assessment. Of these, 24 articles were excluded for the following reasons: 7 were not cohort studies, 12 lacked accessible data, 3 did not report outcomes of interest, and 2 had been superseded by updated publications with larger sample sizes. Consequently, 7 studies (11–17) satisfying the inclusion criteria were included in the meta-analysis. The detailed study selection process is illustrated in **Figure 1**.

### Characteristics and quality assessment of included studies

From 2016 to 2025, a total of seven studies investigated the association between early-life antibiotic exposure and the incidence of T1D in children or adolescents. These studies encompassed more than 7.4 million participants across six countries: Denmark, Finland, Norway, South Korea, Sweden, and the UK. Among these, five studies (12, 14–17) involved antibiotic exposure during pregnancy while six studies (11–14, 16, 17) involved exposure after birth. Detailed characteristics of the included studies are summarized in **Table 1** and **Supplementary Table 1**.

**Table 1:** Characteristics of included studies in the meta-analysis.

Study	Year	Country	Year of birth	No of participants		No of type 1 diabetes		Exposure stage
				Exposure	Non-exposure	Yes	No	
Haupt-Joergensen, M.	2018	Denmark	1997-2003	92,012		336	91,676	During pregnancy
Clausen, T. D.	2016	Denmark	1997-2010	242,419	615,782	1,503	856,698	The first 24 months after birth
Hakola, L.	2025	Finnish	1996-2008	77,132		2,869	74,263	During pregnancy; The first 24 months after birth
Tapia, G.	2018	Norway	2004-2012	537,460		836	536,623	During pregnancy; The first 18 months after birth
Choi, E. Y.	2025	Korea	2009-2020	1,337,837	1,041,734	666	2,378,905	During pregnancy
				1,406,372	1,281,203	745	2,686,803	The first 6 months after birth
Wernroth, M. L.	2020	Swedish	2005-2013	153,741		275	153,466	During pregnancy
				180,410		347	180,063	The first 12 months after birth
Beier, M. A.	2025	UK	1988-2017	68,508	405,706	1974	472,240	The first 24 months after birth

No, number.

The methodological quality of the included cohort studies was evaluated using the NOS. All studies were assessed as high-quality, with scores ranging from 7 to 9. Detailed scoring results are presented in **Supplementary Table 2**.

### Analysis of the primary result

**Figure 2:**
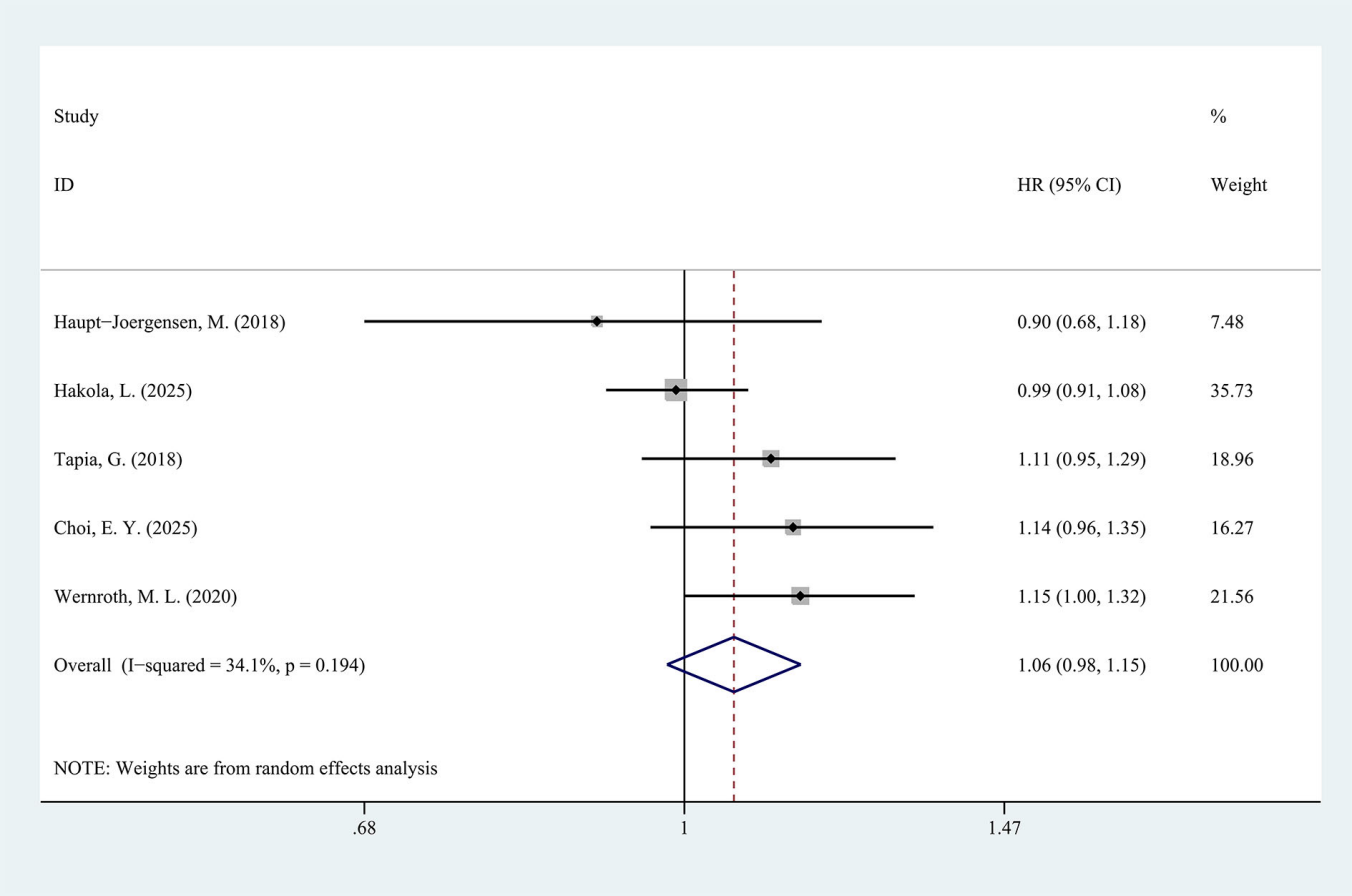
Forest plot of the effect of antibiotic exposure during pregnancy on the incidence of T1D (p=0.146).

A pooled analysis of the five studies examining antibiotic exposure during pregnancy showed no significant differences in the incidence of T1D among children or adolescents between the antibiotic exposure group and the control group (HR = 1.06, 95%CI: 0.98-1.15, p=0.146, **Figure 2**), with moderate heterogeneity observed (*I*^2^ = 34.1%).

**Figure 3:**
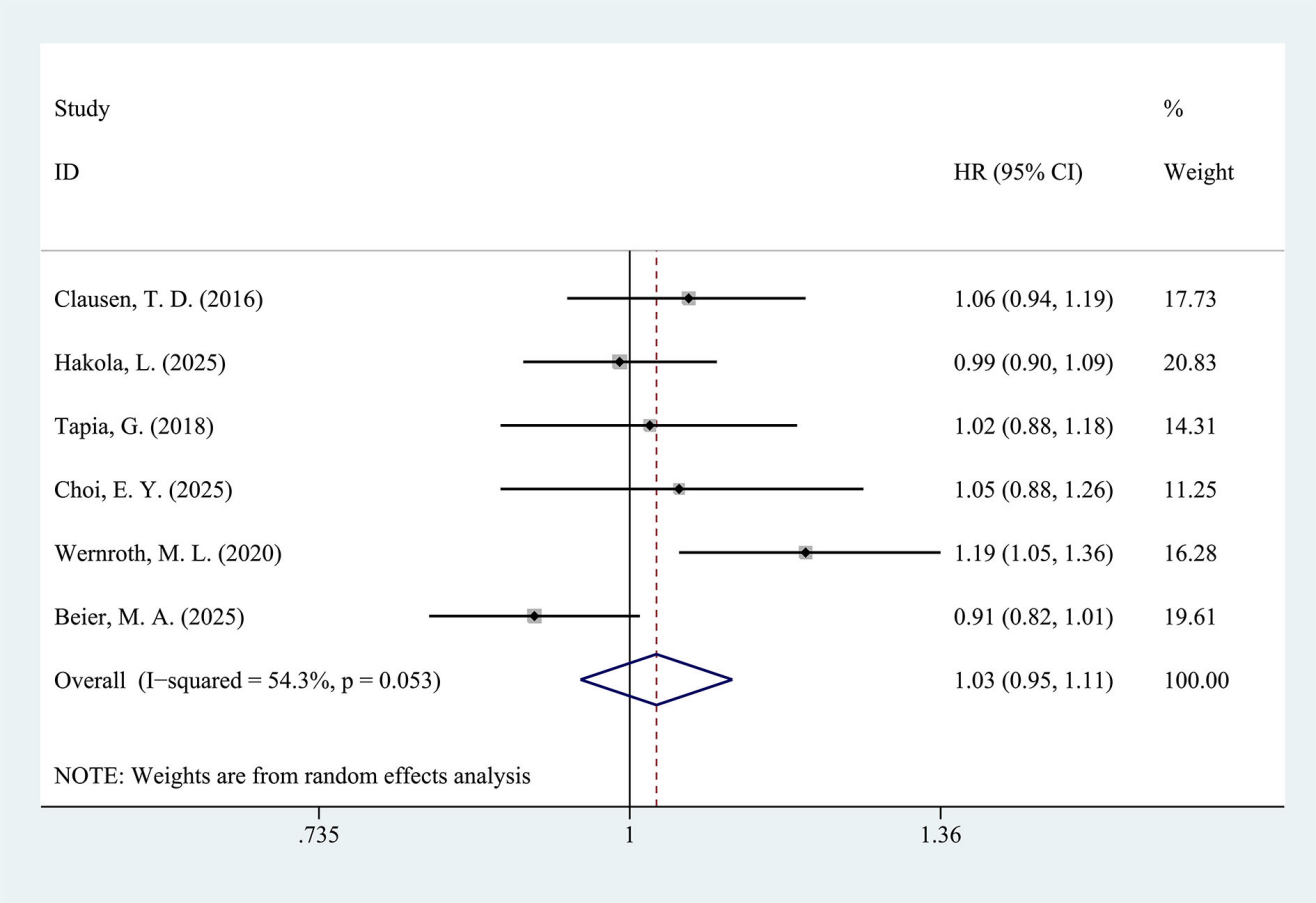
Forest plot of the effect of antibiotic exposure after birth on the incidence of T1D (p=0.491).

Similarly, pooling the results of the six studies assessing antibiotic exposure after birth also revealed no significant differences in T1D risk of children or adolescents between both groups (HR = 1.03, 95%CI: 0.95-1.11, p=0.491, **Figure 3**), although substantial heterogeneity was present in this analysis (*I*^2^ = 54.3%).

### Subgroup analysis

The sibling-matched subgroup analysis for antibiotic exposure during pregnancy demonstrated no significant differences in the incidence of T1D between antibiotic exposure and control groups (HR = 1.07, p=0.588). Additionally, for sex-stratified subgroup analyzes, neither the male group (HR = 1.14, p= 0.109) nor the female group (HR = 1.15, p=0.073) revealed significant differences in T1D rates between two groups. Comprehensive subgroup data are presented in **Table 2**.

**Table 2:** Subgroup analysis of antibiotic exposure during pregnancy.

Subgroup	No. of studies	HR (95% CI)	P	I^2^
Sibling analysis	2	1.07 [0.84, 1.36]	0.588	0%
Gender				
Male	2	1.14 [0.97, 1.33]	0.109	0%
Female	2	1.15 [0.99, 1.33]	0.073	0%
Exposure timing				
1st trimester	3	1.10 [0.94, 1.28]	0.235	14.6%
2nd trimester	3	0.99 [0.81, 1.21]	0.702	0%
3rd trimester	3	1.08 [0.93, 1.24]	0.315	3.8%
Times of exposure				
< 3	3	1.00 [0.93, 1.08]	0.950	0%
≥ 3	2	0.79 [0.28, 2.26]	0.661	61.2%
Types of antibiotics				
β-lactams	4	1.04 [0.92, 1.18]	0.500	22.5%
Cephalosporins	3	1.00 [0.90, 1.11]	0.978	0%
Macrolide, lincosamide and streptogramin	3	1.05 [0.87, 1.26]	0.641	0%
Sulfonamides and trimethoprim	2	1.26 [0.54, 2.91]	0.594	73.9%
Broad-spectrum antibiotics				
Yes	4	1.06 [0.95, 1.18]	0.308	11.0%
No	4	1.07 [0.95, 1.21]	0.281	0%

No, number; HR, hazard ratio; CI, confidence interval.

For antibiotic exposure during pregnancy, further subgroup analyzes were performed based on the exposure timing (1st trimester, 2nd trimester and 3rd trimester), times of exposure (<3 times and ≥3 times), types of antibiotics (β−lactams, cephalosporins, macrolides/lincosamides/streptogramins, and sulfonamides/trimethoprim), and antibacterial spectrum defined by Danish antibiotics categorization DANMAP (narrow and broad-spectrum antibiotics). None of these subgroups showed significant differences between exposed and unexposed group in the incidence of T1D (all p>0.1). More detailed information is provided in **Table 2**.

The same classification criteria including sibling analysis, sex (male and female), exposure timing (the first 6 months of life, the first 1 year of life and the first 2 years of life), times of exposure (<3 times and ≥3 times), types of antibiotics (β−lactams, cephalosporins, macrolides and sulfonamides/trimethoprim) and antibacterial spectrum defined by DANMAP (narrow and broad-spectrum antibiotics) were applied to the analysis of antibiotic exposure after birth. Similarly, no significant differences were found in the incidence of T1D between antibiotics exposure and control groups (all p>0.05). Detailed results are provided **Table 3**.

**Table 3:** Subgroup analysis of antibiotic exposure after birth.

Subgroup	No. of studies	HR (95% CI)	P	I^2^
Sibling analysis	4	1.08 [0.87, 1.34]	0.483	65.1%
Gender				
Male	3	1.10 [0.99, 1.22]	0.069	41.3%
Female	3	1.01 [0.84, 1.23]	0.879	76.6%
Exposure timing				
The first 6 months of life	3	1.06 [0.85, 1.33]	0.599	61.5%
The first 1 year of life	3	1.06 [0.95, 1.19]	0.275	68.2%
The first 2 years of life	3	0.98 [0.90, 1.07]	0.662	45.8%
Times of exposure				
< 3	4	1.05 [0.97, 1.13]	0.257	34.4%
≥ 3	4	1.08 [0.97, 1.21]	0.178	57.2%
Types of antibiotics				
β-lactams	4	1.04 [0.96, 1.12]	0.362	24.0%
Cephalosporins	3	1.08 [0.92, 1.27]	0.356	44.5%
Macrolides	4	0.99 [0.92, 1.08]	0.859	0%
Sulfonamides and trimethoprim	2	0.96 [0.67, 1.37]	0.823	62.9%
Broad-spectrum antibiotics				
Yes	4	1.04 [0.96, 1.13]	0.290	31.8%
No	4	1.07 [0.96, 1.19]	0.229	65.4%

No, number; HR, hazard ratio; CI, confidence interval.

**Figure 4:**
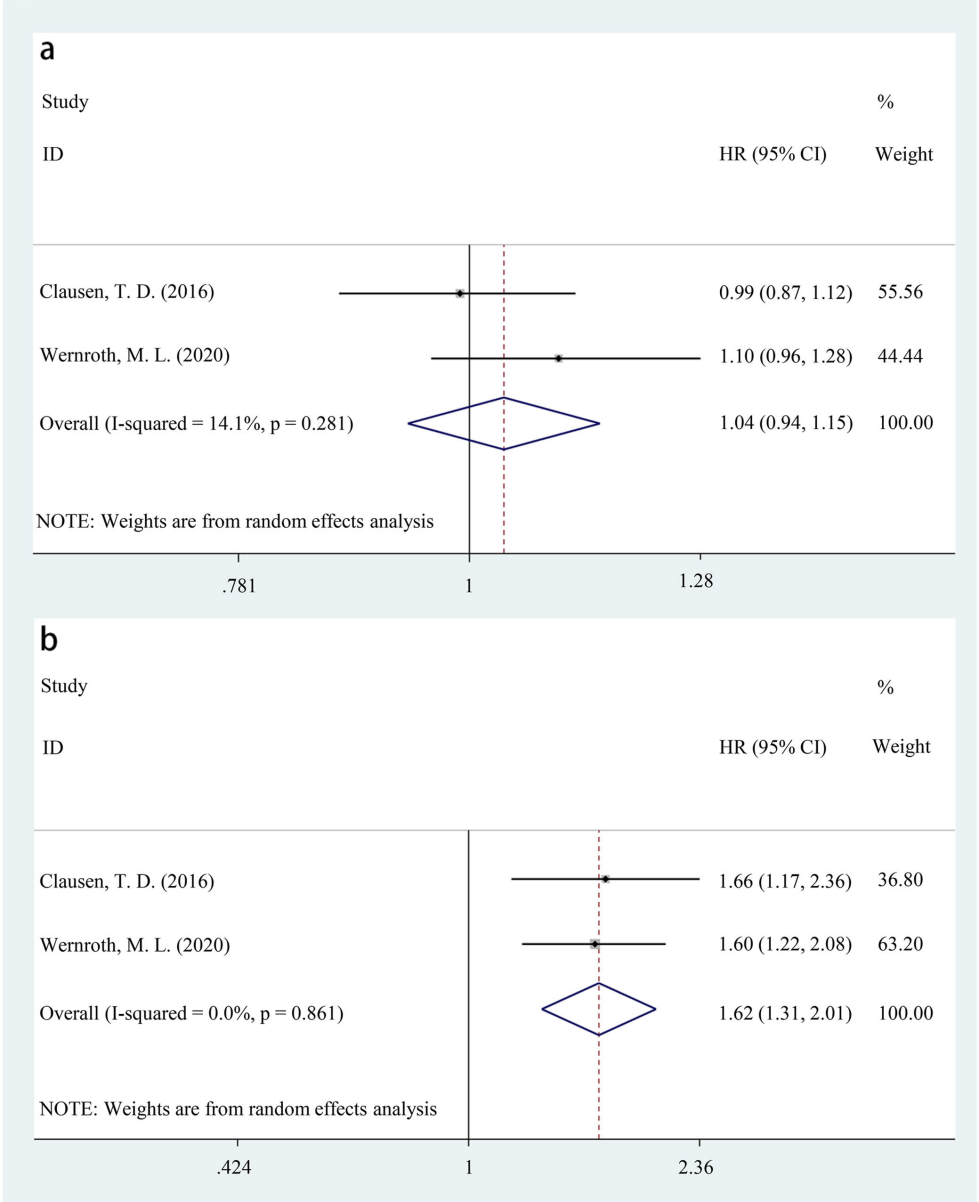
Forest plot of the effect of antibiotic exposure after birth on the incidence of T1D in delivery mode **(a)** vaginally, p=0.482; **(b)** by cesarean section, p<0.001).

Regarding the mode of delivery, for cesarean section, analysis of two studies (13, 17) indicated that antibiotic exposure after birth had a higher incidence of T1D compared to unexposed controls (HR = 1.62, 95%CI: 1.31-2.01, p<0.001). Moreover, there was no heterogeneity detected among these studies (*I*^2^ = 0%). On the contrary, among children delivered vaginally, no significant difference in T1D incidence was found between those exposed to antibiotics after birth and unexposed children (HR = 1.04, 95%CI: 0.94-1.15, p=0.482), with low heterogeneity across studies (*I*^2^ = 14.1%). More detailed information is shown in **Figure 4**.

### Publication bias and sensitivity analysis

Egger’s test revealed no significant publication bias in examining the relationship of T1D with antibiotic exposure, whether during pregnancy (p>0.999, **Supplementary Figure 1**) or after birth (p=0.452, **Supplementary Figure 2**). In addition, sensitivity analysis was performed by sequentially excluding individual studies, with the statistical results remained stable for both exposure analyzes during pregnancy (**Supplementary Figure 3**) and after birth (**Supplementary Figure 4**).

## Discussion

This meta-analysis, which incorporated seven cohort studies, did not reveal a significant association between early-life antibiotic exposure (during pregnancy and after birth) and the incidence of T1D in children or adolescents. The insignificant association remained consistent across the prespecified subgroup analyzes, including sibling comparisons, sex, timing of exposure, frequency of exposure, types of antibiotics, and antibacterial spectrum. However, among individuals delivered by cesarean section, antibiotic exposure after birth might be associated with an increased risk of developing T1D in childhood or adolescence. Notably, the limited number of studies constrains the robustness of this meta-analysis, positioning it as an exploratory study and necessitating a guarded interpretation of its results.

T1D is a T-cell-mediated autoimmune metabolic disorder that primarily occurs in children and adolescents (18). Its core mechanism involves the attack and destruction of pancreatic islet β-cells by activated autoreactive T cells, which subsequently leads to disturbances in blood glucose regulation (18). Evidence from both animal models and human cohort studies indicates a close association between the gut microbiota and the development of islet autoimmunity or the progression of T1D (19, 20). Early life, encompassing both prenatal and postnatal periods, is a critical developmental phase for the colonization, succession, and stabilization of the gut microbiota, which are processes integrally linked to host immune maturation (21). Research has suggested that antibiotic use during pregnancy perturbs the maternal microbiome and may vertically transmit this state of dysbiosis to the offspring (22). Furthermore, longitudinal studies tracking the natural development of the infant gut microbiota have revealed that postnatal antibiotic exposure leads to a sharp decline in bacterial strain diversity and disrupts the structural stability of the microbial community (23). Acting as a central alteration, gut microbiota dysbiosis may influence the onset and progression of islet autoimmunity through several interconnected mechanisms.

Compromised intestinal barrier integrity represents one key pathway (24). Studies indicated that gut microbiota dysbiosis can induce intestinal barrier dysfunction, primarily by increased paracellular permeability, reduced expression of tight junction proteins, and disrupted morphology of zonula occludens (25). Concurrently, gut microbiota dysbiosis may contribute to impaired barrier function by activating the NLRP3 inflammasome and autophagy pathways (25). This impairment of barrier function allows luminal metabolites, dietary antigens, or infectious agents to translocate from the gut lumen to mucosal immune components. Such translocation can directly trigger systemic low-grade inflammation, potentially activating islet-reactive T cells (26). Alternatively, through molecular mimicry with islet autoantigens, these substances leaked from the intestines may induce cross-reactive T cells that subsequently damage islet β-cells (27).

Additionally, gut microbiota dysbiosis can contribute to a shift in T-cell subset balance toward pro-inflammatory phenotypes. The equilibrium tilts in favor of pro-inflammatory T cells such as Th1 and Th17, resulting in elevated levels of inflammatory cytokines including interleukin-17 (IL-17) and interferon-γ, alongside a decrease in suppressive cytokines like IL-10 and transforming growth factor-β (5). This shift undermines immune tolerance and promotes an opportunity for autoimmunity to attack islet β-cells.

Furthermore, gut microbiota dysbiosis facilitates the trafficking of immune cells to the pancreas. The migration of islet-reactive T cells from pancreatic lymph nodes into the islets is mediated by specific chemokine receptor-ligand interactions (28). Studies have confirmed that gut microbiota dysbiosis upregulates both the T-cell receptor C-X-C chemokine receptor type 3 (CXCR3) and its corresponding ligand C-X-C motif chemokine ligand 10 (CXCL10) within pancreatic islets. This coordinated increase enhances the chemotactic recruitment of T cells to the islets (29), thereby exacerbating islet autoimmunity and advancing the pathogenesis of T1D.

Gut microbiota dysbiosis further contributes to disease pathogenesis by altering the microbial metabolome. Key metabolites, including short-chain fatty acids such as acetate, propionate, and butyrate, are produced from bacterial fermentation of dietary fiber (30). These compounds exert anti-inflammatory effects, promote regulatory T cell differentiation, and enhance intestinal barrier integrity (30). Additionally, bacterial metabolism of tryptophan generates indole derivatives like indolelactic acid and indoleacetic acid, which stimulate IL-22 production to support mucosal integrity and dietary antigen tolerance (31). Conversely, antibiotic exposure reduces levels of these beneficial metabolites, compromises immune tolerance, and may thereby promote immune-mediated destruction of pancreatic β-cells.

In addition to the direct consequences of intestinal dysbiosis, animal studies have confirmed that antibiotic exposure during pregnancy can also impair immune development in the offspring and increase the risk of immune-related disorders not only through the disruption of maternal immune homeostasis extending to the placental interface (32), but also via the down-regulation of FcRn receptor expression in mammary tissue, which lowers the concentration of IgG in colostrum and thus weakens the immune protection transferred from the mother (33).

While the preceding mechanisms provide a theoretical basis for antibiotic exposure increasing the risk of T1D, our findings align with previous meta-analyzes in demonstrating no significant association. Several factors may explain this apparent discrepancy. (1) The Swedish ABIS study (34) revealed that the relationship between antibiotic use and disease exhibited entirely distinct patterns across groups with different genetic risks. As most participants in the present meta-analysis were drawn from the general population, the effect of early-life antibiotic exposure may be significant only against specific genetic backgrounds, such as in carriers of high-risk HLA genes, whereas its impact is diluted in unselected populations. (2) The progression of T1D involves a multi-stage process from single to multiple autoantibody positivity, and ultimately to clinical onset (35). Antibiotic exposure in early life may primarily influence the initial stages of disease development. This effect can be obscured by various confounding factors, including the baseline microbiota, diet, and environmental exposures, which may explain why meta-analyzes using clinical diagnosis as the endpoint did not detect a significant association. (3) The infant gut microbiota exhibits a degree of repairability. Consequently, short-term or single-course antibiotic exposure may induce only transient and reversible changes that are insufficient to cause the permanent alterations required to trigger sustained autoimmunity. (4) The meta-analysis was constrained by limited sample sizes, which affected the reliability of the results.

The present meta-analysis provided the first reported association between antibiotic exposure after birth and an elevated risk of T1D in children delivered by cesarean section. The following mechanisms may help explain this observation. (1) Cesarean delivery has been associated with increased DNA methylation levels in offspring blood, thereby altering epigenetic profiles (36). DNA hypermethylation has been implicated in the pathogenesis of several autoimmune diseases, including T1D, rheumatoid arthritis, Sjögren’s syndrome, Graves’ disease, and Crohn’s disease (37). (2) Cesarean section can delay or reduce the colonization of beneficial gut microbes such as Lactobacillus and Bifidobacterium, which are thought to help protect against T1D development (38). (3) Cesarean birth may upregulate dopamine D1 receptor expression and enhance functional responses in offspring (39). Signaling through this receptor promotes pathogenic Th17 cell differentiation and the production of the pro-inflammatory cytokine IL-17 (40), potentially initiating an inflammatory cascade that contributes to autoimmune islet destruction. (4) Gene expression profiles in peripheral blood mononuclear cells show systematic similarities between children delivered by cesarean section and those who develop islet autoimmunity. These similarities are characterized by altered activity in immune cell activation pathways, particularly those involving the pentose phosphate pathway and pyrimidine metabolism (41), which may predispose the immune system to aberrant activation. (5) Vaginal delivery exposes the infant to intermittent mechanical forces, transient hypoxia, oxidative stress, and hormonal responses to maternal stress such as adrenaline and cortisol (42). These exposures serve as important developmental signals for multiple physiological systems (42). The absence of such stimuli in cesarean-born infants may impair immune system maturation, increasing susceptibility to autoimmune disorders. Taken together, these underlying risk factors associated with cesarean section may synergize with antibiotic exposure after birth, ultimately contributing to the development of T1D. By the way, a meta-analysis (43) has established cesarean delivery itself as a risk factor for T1D. However, due to the limited data, the result should be interpreted with caution.

Existing meta-analyzes (7, 8) examining antibiotic exposure during pregnancy or after birth used odds ratios (ORs) to evaluate the association and lacked further subgroup analyzes. In contrast, the current meta-analysis employed HRs as the effect measure, with stronger reliability of the results. Moreover, this study performed extensive pre-specified subgroup analyzes based on sibling design, sex, exposure timing, frequency of exposure, types of antibiotics, and antibacterial spectrum and results remained stable, providing a more comprehensive and reliable evaluation of the association between early-life antibiotic exposure and T1D.

Although the included studies were of high quality, the overall certainty of evidence was rated as low according to the GRADE framework. Several limitations should be considered. Firstly, numerous potential confounders like ethnicity, family history, feeding patterns, dietary habits and socioeconomic status might distort the true relationship between antibiotic use and T1D, which in turn lowered confidence in the evidence. Secondly, antibiotic exposure was inherently linked to underlying infections, making it difficult to isolate the independent effect of antibiotics from that of the infection itself, which affected the reliability of the findings. This concern is compounded by evidence suggesting that early-life infections may increase the incidence of T1D among children without genetic susceptibility to the disease, further blurring the causal pathway (34). Thirdly, the inadequate data also constituted the important limitations.

This meta-analysis may support the development of more tailored recommendations for antibiotic use in specific high-risk pediatric populations. In clinical practice, a history of cesarean delivery may serve as a potential risk-stratification marker when prescribing antibiotics to children. Furthermore, future antibiotic safety research and clinical guidelines should pay closer attention to high-risk subgroups including children born by cesarean section.

## Conclusion

The significant association between early-life antibiotic exposure (during pregnancy and after birth) and the incidence of T1D among general children or adolescents was not observed in the meta-analysis. However, for children born by cesarean section, antibiotic exposure after birth might increase the risk of T1D. Nevertheless, these findings warrant further validation in larger, well-designed studies.

## Supplementary material

Supplementary Figure 1 Egger’s test for assessing publication bias of included studies involving antibiotic exposure during pregnancy (p>0.999).

Supplementary Figure 2 Egger’s test for assessing publication bias of included studies involving antibiotic exposure after birth (p=0.452).

Supplementary Figure 3 Sensitivity analysis for testing the stability of included studies involving antibiotic exposure during pregnancy.
