# Supplementary figures and images for: The association between early-life (during pregnancy and after birth) antibiotic exposure and type 1 diabetes: an updated meta-analysis
Source: Front Endocrinol (Lausanne). 2026 Apr 22;17:1807564. doi: 10.3389/fendo.2026.1807564 (PMC13143605; doi:10.3389/fendo.2026.1807564)

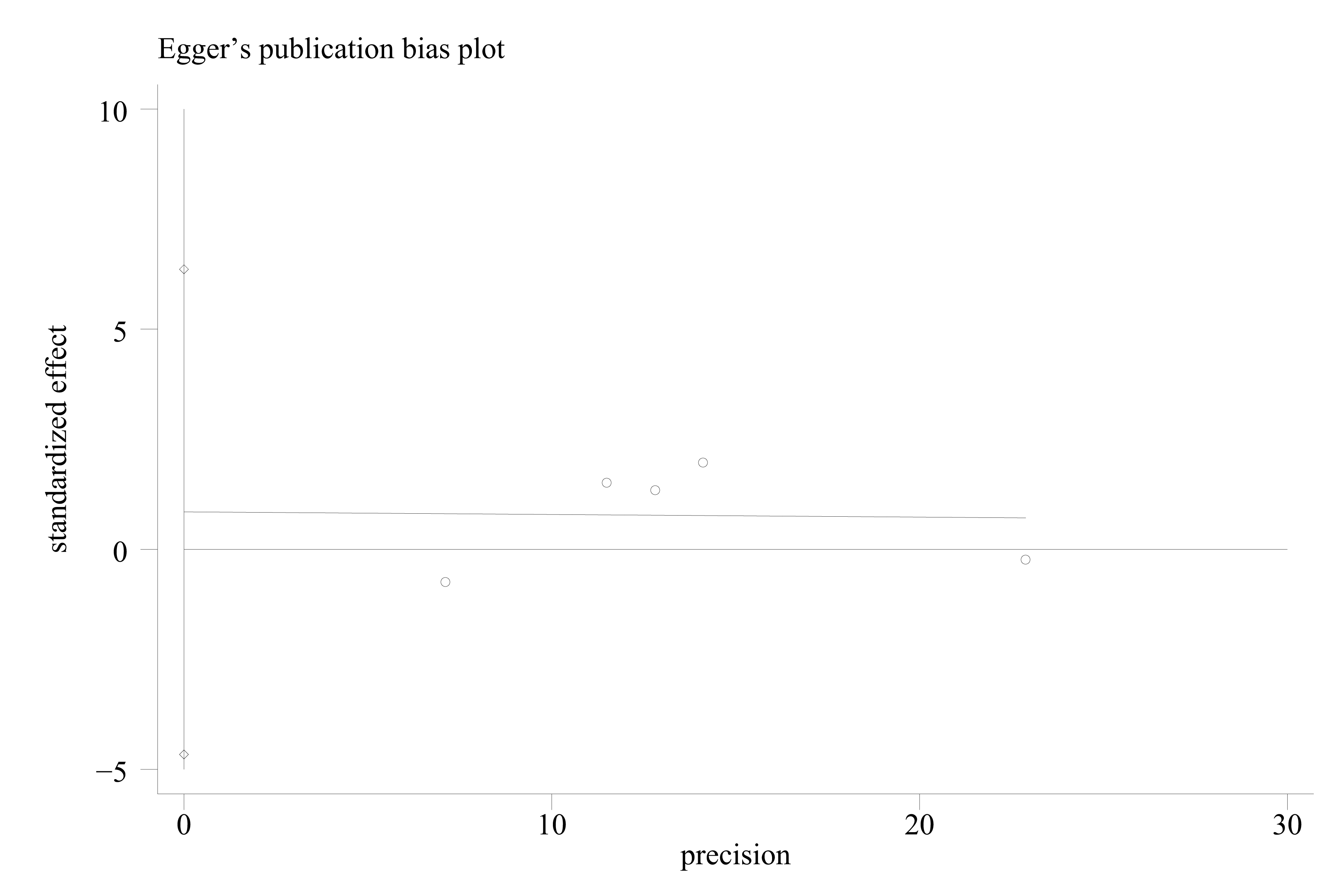

Supplement: Supplementary Figure 1 — Egger’s test for assessing publication bias of included studies involving antibiotic exposure during pregnancy (p>0.999). [file Image1.tif]

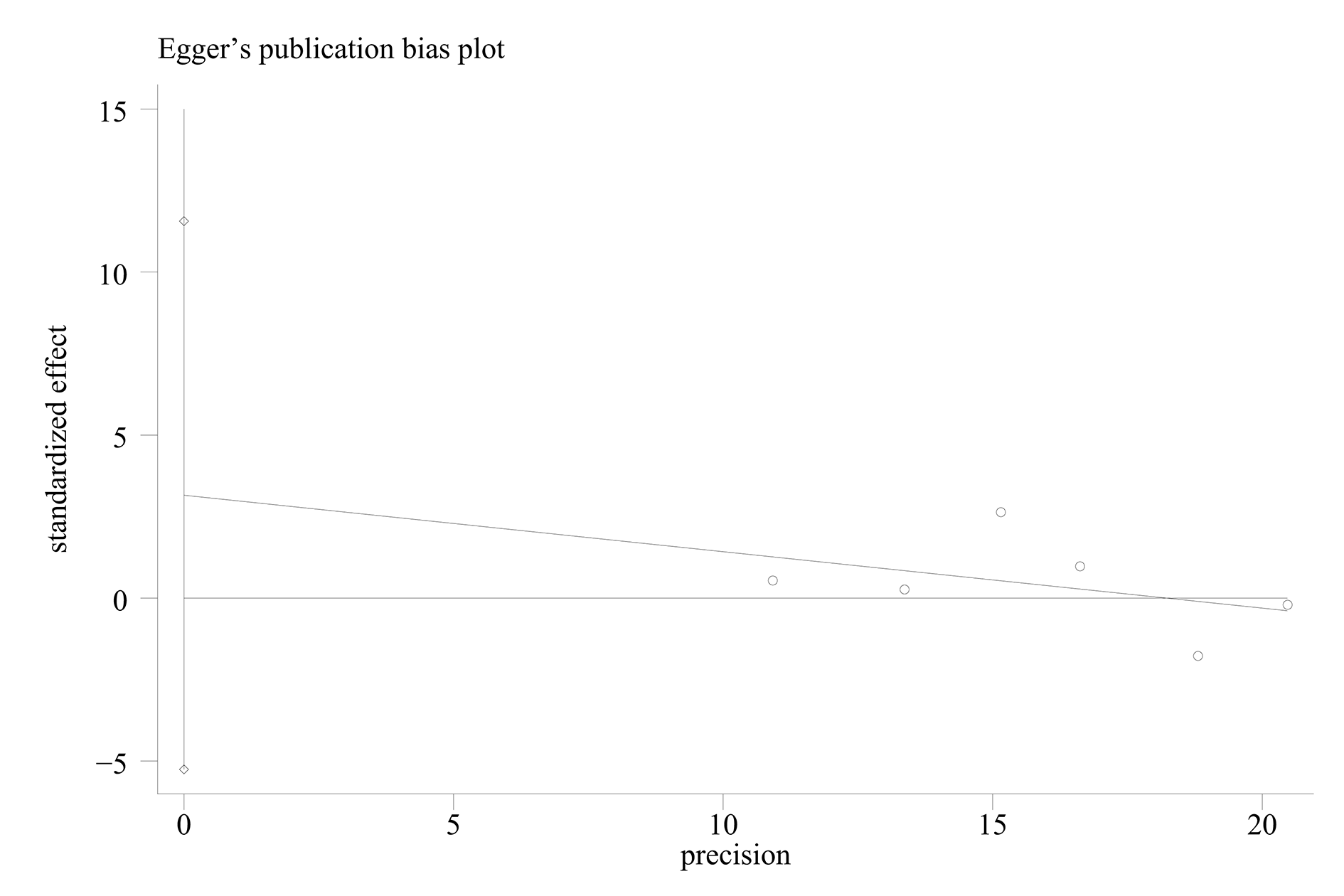

Supplement: Supplementary Figure 2 — Egger’s test for assessing publication bias of included studies involving antibiotic exposure after birth (p=0.452). [file Image2.tif]

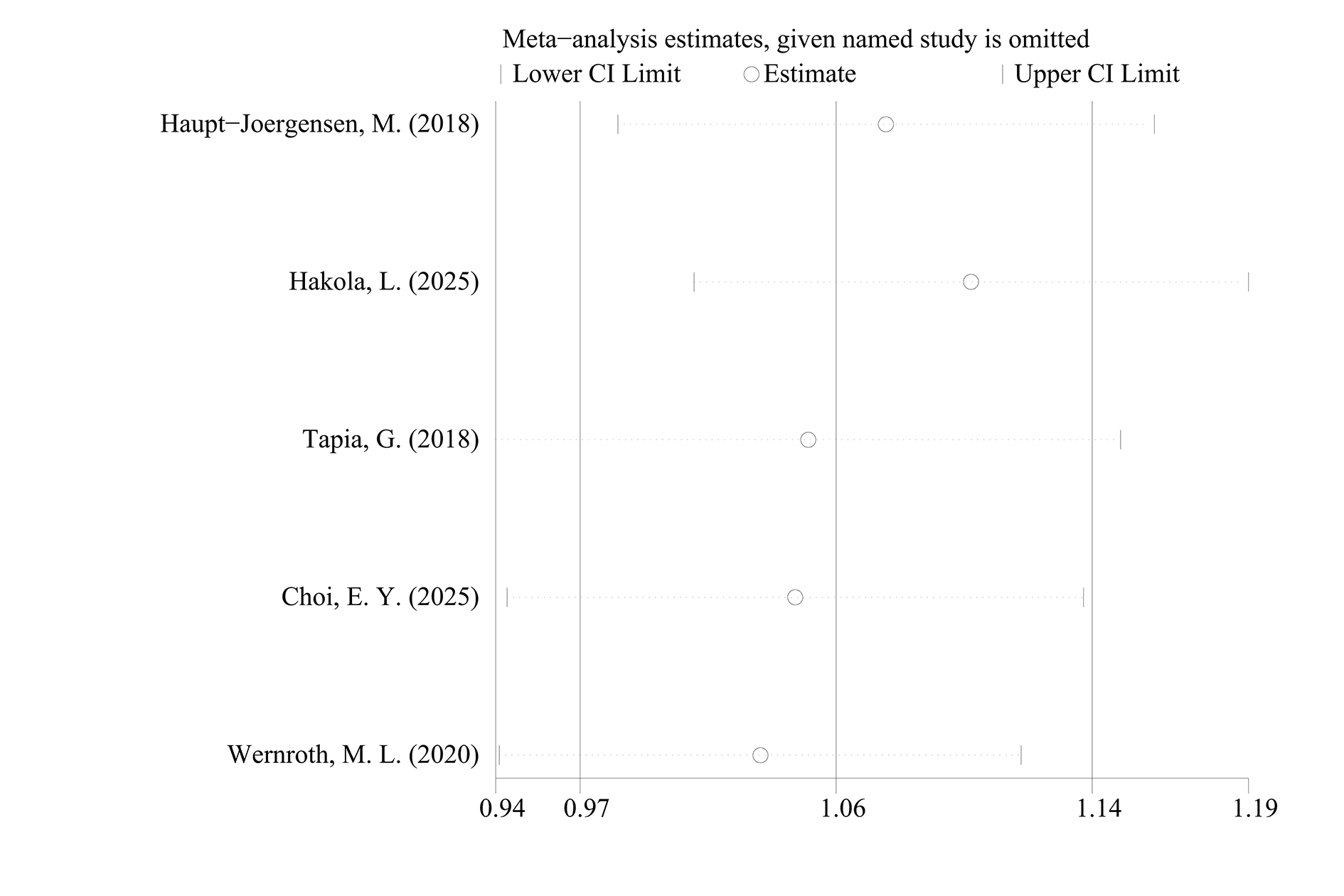

Supplement: Supplementary Figure 3 — Sensitivity analysis for testing the stability of included studies involving antibiotic exposure during pregnancy. [file Image3.tif]

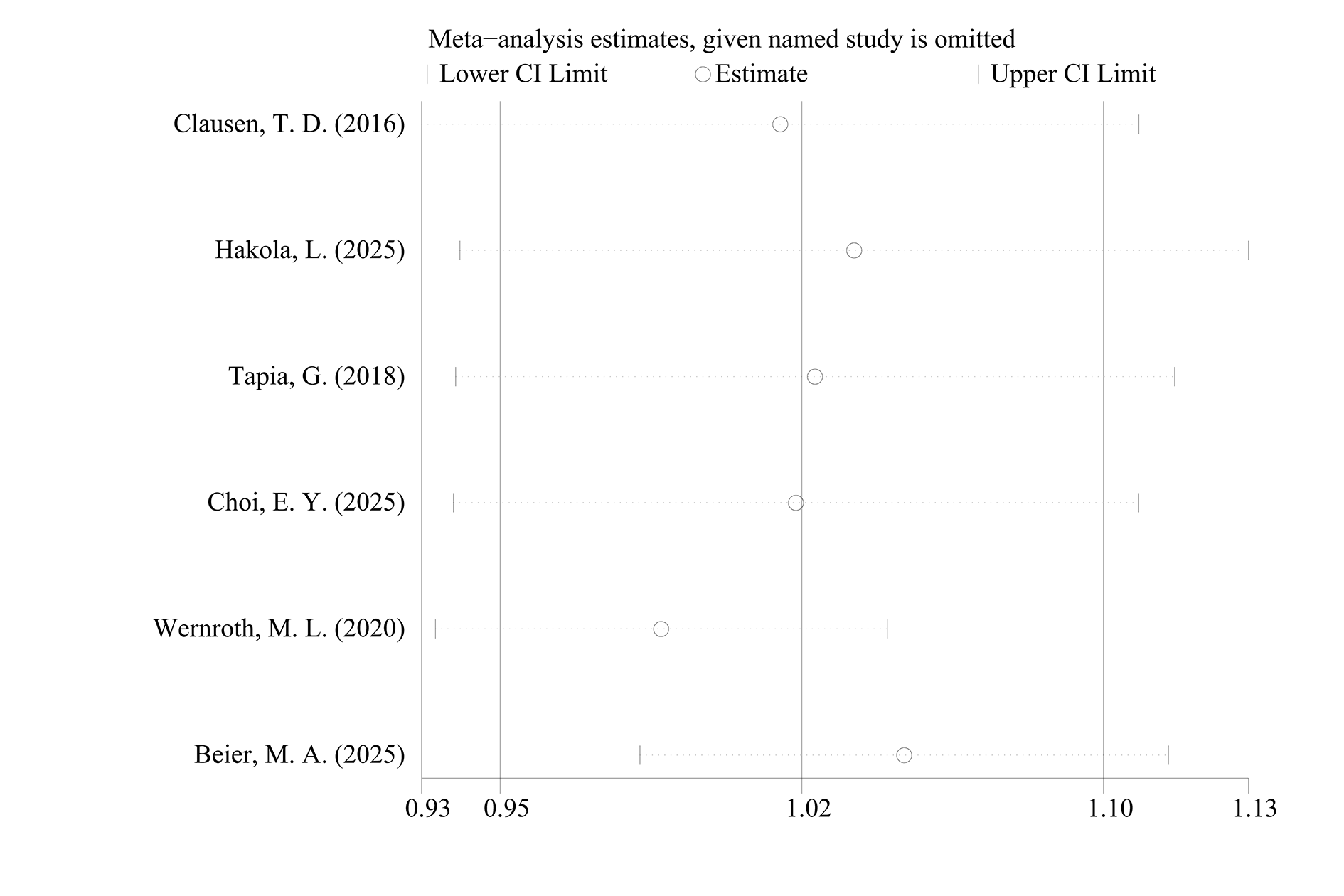

Supplement: Supplementary file 4 [file Image4.tif]
