# Supplementary material for: The association between early-life (during pregnancy and after birth) antibiotic exposure and type 1 diabetes: an updated meta-analysis
Source: Front Endocrinol (Lausanne). 2026 Apr 22;17:1807564. doi: 10.3389/fendo.2026.1807564 (PMC13143605; doi:10.3389/fendo.2026.1807564)
Supplement: Supplementary file 5 [file Table1.docx]

**Supplementary Table 1. Characteristics of included studies in the meta-analysis.**

| Author | Year | Factors adjusted for |
| --- | --- | --- |
| Haupt-Joergensen, M. | 2018 | maternal prepregnancy BMI, paternal BMI, maternal age, socioeconomic status, parity, maternal diabetes, maternal smoking during pregnancy, birth weight, and gestational weight gain. |
| Clausen, T. D. | 2016 | year of birth, mode of delivery, sex, parity, maternal age, paternal age, parental education level, maternal type 1 diabetes, and paternal type 1 diabetes. |
| Hakola, L. | 2025 | maternal age, maternal smoking during pregnancy, maternal diabetes, maternal asthma, parity, year of birth, season of birth, mode of delivery, sex, gestational age, birth weight, and childhood asthma. |
| Tapia, G. | 2018 | parity, sex, maternal age, maternal type 1 diabetes, parental education level, prematurity, birth weight, and mode of delivery. |
| Choi, E. Y. | 2025 | maternal age, maternal BMI, maternal smoking during pregnancy, insurance type, income level, year of delivery, cause of infections, proxies for severity of infection, maternal comorbidities, concomitant medications, healthcare utilization, nulliparity, multiple gestations, obstetric comorbidity index, sex, maternal exposure to antibiotics, prematurity, cesarean section, birth weight, and type of feeding. |
| Wernroth, M. L. | 2020 | parity, maternal smoking during early pregnancy, maternal type 1 diabetes, maternal age, parental country of birth, parental education level, income level, year of birth, season of birth, region of residence, population density, maternal BMI, mode of delivery, sex, gestational age, paternal type 1 diabetes, and small or large for gestational age. |
| Beier, M. A. | 2025 | maternal comorbidities, concomitant medications, prenatal and perinatal factors, infections during pregnancy and early childhood, healthcare utilization, and area-based socioeconomic status. |

BMI, body mass index.
