# Supplementary material for: The association between early-life (during pregnancy and after birth) antibiotic exposure and type 1 diabetes: an updated meta-analysis
Source: Front Endocrinol (Lausanne). 2026 Apr 22;17:1807564. doi: 10.3389/fendo.2026.1807564 (PMC13143605; doi:10.3389/fendo.2026.1807564)
Supplement: Supplementary file 6 [file Table2.doc]

**Supplementary Table 2. Quality assessment of the** cohort studies included.

| Author, year | **Selection (Out of 4)** | | | | **Comparability**  **(Out of 2)** | **Outcomes (Out of 3)** | | | **Total**  **(Out of 9)** |
| --- | --- | --- | --- | --- | --- | --- | --- | --- | --- |
|  | Representativeness of exposed cohort | Selection of non exposed cohort | Ascertainment  of exposure | Outcome not present at the start of the study |  | Assessment of outcomes | Length of follow-up | Adequacy of follow up of cohorts |  |
| Haupt-Joergensen, M,2018 | 1 | 1 | 1 | 1 | 1 | 1 | 1 | 1 | 8 |
| Clausen, T. D,2016 | 1 | 1 | 1 | 1 | 1 | 1 | 1 | 0 | 7 |
| Hakola, L,2025 | 1 | 1 | 1 | 1 | 2 | 1 | 1 | 0 | 8 |
| Tapia, G,2018 | 1 | 1 | 1 | 1 | 1 | 1 | 1 | 0 | 7 |
| Choi, E. Y,2025 | 1 | 1 | 1 | 1 | 2 | 1 | 0 | 0 | 7 |
| Wernroth, M. L,2020 | 1 | 1 | 1 | 1 | 2 | 1 | 1 | 0 | 8 |
| Beier, M. A,2025 | 1 | 1 | 1 | 1 | 2 | 1 | 1 | 1 | 9 |

The cohort studies were assessed by the Newcastle-Ottawa Quality Assessment Scale (NOS) checklist.
